# Molecular Dynamics Research on the Impact of Vacancies on Cu Precipitation in BCC-Fe

**DOI:** 10.3390/ma14175029

**Published:** 2021-09-02

**Authors:** Haichao Zhang, Yinli Chen, Xufeng Wang, Huirong Li, Yungang Li

**Affiliations:** 1 Department College of Metallurgy and Energy, North China University of Science and Technology, Tangshan 063210, China; d202110611@xs.ustb.edu.cn (H.Z.); Wangxufeng@stu.ncst.edu.cn (X.W.); lihuirong@ncst.edu.cn (H.L.); 2 Department Collaborative Innovation Center of Steel Technology, University of Science and Technology Beijing, Beijing 100089, China; yinli_chen@ustb.edu.cn

**Keywords:** molecular dynamics, Cu cluster, structural phase transition, vacancy element

## Abstract

The molecular dynamics (MD) simulation method was used to explore the impact of vacancy concentration (0 at%, 0.1 at% and 0.2 at%) on the diffusion and precipitation rate of Cu atoms in the Fe-3.5Cu alloy and the growth of Cu precipitation during the aging process of the alloy. The mechanism of the influence of Cu precipitation relative to the tensile properties of Fe-3.5Cu alloy was investigated. The results showed that the presence of vacancies will promote the diffusion and precipitation of Cu atoms in the Fe-3.5Cu alloy, but the diffusion and precipitation rate of Cu atoms does not always increase with the increase in vacancies. In the alloy containing 0.2 at% vacancies, the diffusion and precipitation rate of Cu atoms is lower than that in the alloy containing 0.1 at% vacancies. During the aging process, when the alloy contains no vacancies, no Cu precipitates will be produced. In the alloy containing 0.1 at% vacancies, the size of the Cu precipitates produced is larger than the size of the Cu precipitates produced in the alloy containing 0.2 at% vacancies, but the number of precipitates is less than that in the alloy with 0.2 at% vacancies. During the tensile process, the Cu precipitates will promote early occurrence of phase transition of the internal crystal structure in the Fe-3.5Cu alloy system, and lead to the generation of vacancy defects in the system, thus weakening the yield strength and strain hardening strength of the alloy.

## 1. Introduction

High-strength low-alloy steel (HSLA) has been widely used in the field of shipbuilding because of its advantages, such as high strength, good toughness and easy welding [1,2]. For example, HSLA steel is used in some structural materials of Arleigh Burke-class guided missile destroyers and Nimitz-class nuclear-powered aircraft carriers currently in service in the United States. Studies have shown that the excellent properties of HSLA steel are mainly attributed to its ultra-low carbon and high-copper alloy design. Under the condition of ensuring good toughness, the ε-Cu phase dispersion and precipitation strengthening during the aging process can compensate for the loss of strength caused by the reduced C content, while reducing the welding crack susceptibility [3,4,5,6]. As an inherent defect of alloy materials, vacancy will have an important impact on the diffusion and precipitation of doped elements in alloys [7,8]. Thus, it is very important to understand the effect of vacancy on Cu precipitation behavior in HSLA steel during the aging process, in order to improve the mechanical properties of HSLA steel and to design and develop new high-quality HSLA steel grades. Lv et al. [9] studied the effect of vacancy in BCC (body-centered cubic)-Fe on Cu atom precipitation during aging by using the molecular dynamics (MD) method. They found that the presence of vacancies will expand the diffusion coefficient of Cu atoms and promote the formation of Cu precipitates containing twin boundary and stacking faults of the FCC structure. Li et al. [10] used the MD method to calculate the segregation energy of Cu atoms at the BCC-Fe grain boundary. The calculation results showed that when there are vacancies at the grain boundary, the segregation energy of Cu atoms is smaller than that of Cu atoms when there are no vacancies at the grain boundary. The results indicate that vacancy can promote segregation of Cu atoms towards grain boundaries during aging. Zhang et al. [11] calculated the binding energy between Cu atoms and vacancies, and between Cu atoms in BCC-Fe using MD. It was found that the binding energy between Cu atoms and vacancies is greater than that between Cu atoms, suggesting that when there are vacancies in the system, Cu atoms will preferentially bind to the vacancies, rather than binding to other Cu atoms, and vacancies may play the role of nucleation particles in the growth of the Cu precipitates. Dump et al. [12] adopted HREM (High Resolution Electron Microscopy) to investigate the phase change of Cu-rich precipitates in reactor pressure vessel (RPV) steel under both aging and radiation conditions and found that the critical size of the phase change of Cu-rich precipitates under aging conditions is greater than that under radiation conditions. The analysis showed that the large number of vacancies generated in the RPV steel under the radiation condition led to a reduction in the critical size of the phase transition of Cu-rich precipitates under radiation conditions.

Regarding the existing research on the effect of vacancies on the precipitation of Cu in steel, the specific mechanism of the influence of vacancy content in steel on the growth of Cu precipitates and the mechanism of the changes in mechanical properties of Fe–Cu alloys after aging have rarely been reported. The MD method is based on the nanoscale research method, which can reveal the microscopic mechanism of the change of mechanical properties of materials at the atomic level. Compared with the traditional experimental research method, the molecular dynamics simulation research method can shorten the research time and reduce the research cost.

**Figure 1:**
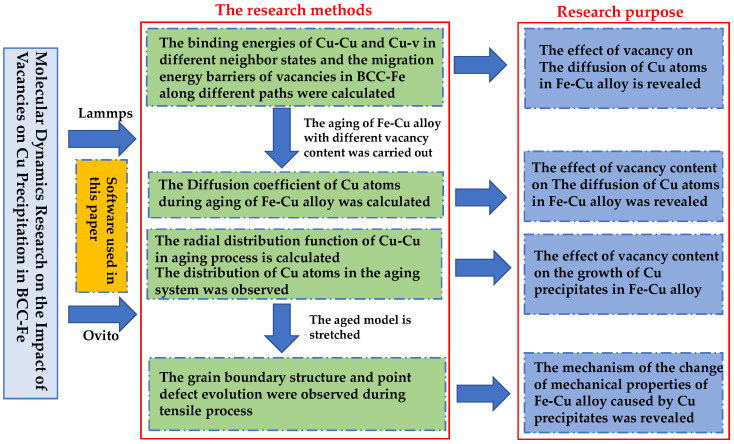
The technology roadmap.

Therefore, based on the existing research, this work aims to use the MD method to analyze how the vacancy content in BCC-Fe affects the precipitation of Cu and the mechanism of the impact of Cu precipitates on the tensile properties of the alloy, so as to lay a theoretical foundation for the development of higher quality HSLA steel for shipbuilding. The technical route studied in this paper is shown in Figure 1. First, the mechanism of the effect of vacancy content (0 at%, 0.1 at% and 0.2 at%) on the precipitation of Cu in BCC-Fe was studied by calculating the atomic binding energy, migration energy barrier, diffusion coefficient and radial distribution function. Then, the mechanism of the impact of Cu precipitates on the tensile properties of the Fe–Cu alloy was revealed through the observation and analysis of the crystal microstructure and point defect evolution during the tensile process.

## 2. Simulation Method

Based on the research content of this work, Atomsk [13] software (V. beta-0.11, 2021, University of Lille, Lille, France) was used firstly to build Fe-3.5Cu-0V (vacancy), Fe-3.5Cu-0.1V and Fe-3.5Cu-0.2V alloy models. In the simulation, the alloy model used was a 42.996 Å × 42.996 Å × 42.996 Å simulation box containing about 6750 atoms. Subsequently, the MD method was used for simulation, and the large-scale atomic/molecular massively parallel simulator (LAMMPS Stable Release, 2020, Sandia National Laboratories, Livermore, CA, USA) [14] was adopted for simulation calculation. The interaction potential function developed by Bonny et al. [15] was used for the simulation calculations. This potential function system has been used to study the interaction between Cu precipitates and point defects and dislocation in Fe–Cu alloys in the past few years, and it can accurately describe the relationship between Cu atoms and defects in Fe–Cu alloys [16,17,18]. Finally, the simulation results were visualized by OVITO [19] visualization software (OVITO Basic, 2021, Darmstadt University of Technology, Hesse-Darmstadt, Germany), and the common neighbor analysis and Wigner–Seitz defect analysis methods were employed to analyze the crystal microstructure and point defect evolution in the simulated tensile process.

**Figure 2:**
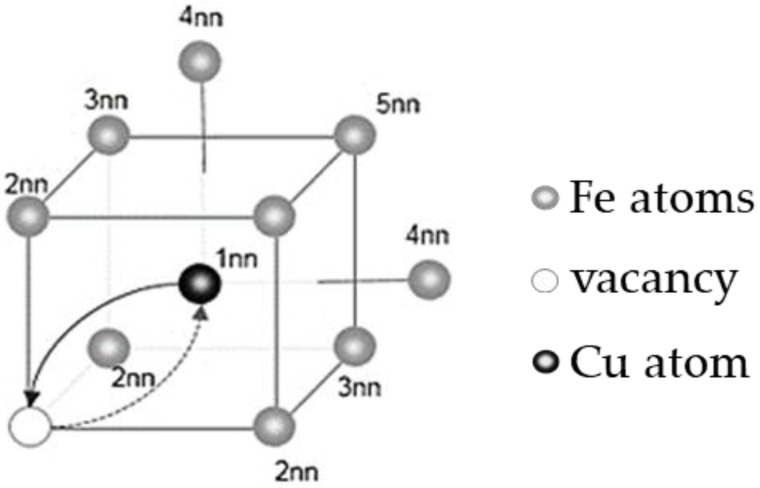
Schematic diagram of atomic neighboring state and migration path.

The impact of vacancies in the Fe–Cu alloy on the diffusion of Cu atoms was illustrated by calculating the binding energy of Cu–Cu and Cu–V in different neighboring states and the migration energy barrier of vacancies when they migrate along different paths in BCC-Fe. The binding energy was calculated by Equation (1) [20], where $E_{b_{inn}}^{\mathrm{Cu}-V}$ is the binding energy of Cu–V in the i-th neighboring state (i = 1, 2, 3, 4 and 5, the neighboring state is shown in Figure 2), N is the total number of atoms in the model, E(NFe), E((N−1)Fe+1Cu), E((N−1)Fe+1V) and E((N−2)Fe+1V+1Cu) respectively represent the total energy of a perfect BCC-Fe system, the total energy of the system with one Cu atom, the total energy of the system with one vacancy, and the total energy of the system with one Cu atom and one vacancy. According to the definition of binding energy and Formula (1), when the binding energy is a positive value, it indicates mutual attraction between atoms, and a negative value means mutual repulsion between atoms. Then, the NEB (nudged elastic band) method was used to calculate the migration energy barrier of vacancies when migrating along different paths in BCC-Fe. Figure 2 shows the migration paths of the vacancy. NEB is a method used to find the minimum energy path of saddle point with known reactants and products, and it can be used for the calculation of the diffusion path, diffusion energy barrier and transition state. When the vacancy moves along a certain path, the smaller the migration energy barrier, the easier it is for the vacancy to migrate along this path. Otherwise, the vacancy is not easy to migrate along this path.

(1) $$E_{b_{inn}}^{\mathrm{Cu}-V}=E\left(\left(N-1\right)\mathrm{Fe}+1\mathrm{Cu}\right)E+E\left(\left(N-1\right)\mathrm{Fe}+1V\right)-E\left(N\mathrm{Fe}\right)-E\left(\left(N-2\right)\mathrm{Fe}+1V+1\mathrm{Cu}\right)$$

The diffusion coefficient of Cu atoms in the system during the aging process of the Fe-3.5Cu-0V, Fe-3.5Cu-0.1V and Fe-3.5Cu-0.2V alloys was calculated to further explain the influence of vacancies in Fe–Cu alloys on the diffusion and precipitation of Cu atoms, and to reveal the impact of the content of vacancies in the alloy on the diffusion and precipitation of Cu atoms. The mean square displacement (MSD) [21] method was adopted to calculate the diffusion coefficient, as shown in Equation (2). The specific steps and parameters of the simulated aging process are as follows: first, the energy of the model built by the Atomsk software was minimized; then the model was relaxed for 200 ps at 1050 K under NVT (canonical ensemble) ensemble; finally, the relaxed model was subjected to an isothermal aging for 200 ns under the same temperature and ensemble, and the simulation time step was 0.002 ps.

(2) $$\mathrm{MSD}=\langle r^{2}\left(t\right)\rangle =\frac{1}{N}\overset{N}{\underset{i=1}{\sum }}\langle {\left|r\left(t\right)-r\left(0\right)\right|}^{2}\rangle$$

The diffusion coefficient can be expressed by the Einstein Equation (3) [22]:

(3) $$D^{*}=\underset{t\to \infty }{\lim}\frac{1}{2Nt}\langle {\left|r\left(t\right)-r\left(0\right)\right|}^{2}\rangle$$

The combination of Equations (2) and (3) leads to:

(4) $$D^{*}=\frac{MSD}{2Nt}$$

D*: diffusion coefficient;

*N*: the dimension of the simulated system, *N* = 3 in this study;

*t*: the simulation time;

r(t), r(0): the position of the atom at time t and the initial position of the atom.

By observing and analyzing the Cu atom distribution and Cu–Cu radial distribution function during the aging process of Fe-3.5Cu-0V, Fe-3.5Cu-0.1V and Fe-3.5Cu-0.2V alloys, the impact of the concentration of vacancies in the Fe–Cu alloy on the growth of Cu precipitates was explored. According to the physical meaning of the radial distribution function, the higher the peak value of the radial distribution function, the greater the probability that another atom exists around an atom [22]. Taking Cu–Cu as an example, it is the ratio of the probability of finding another Cu atom to the probability of random distribution at the radius, r, from the Cu atom as the base point atom. The larger the ratio, the more Cu atoms exist around the base point Cu atom, and the smaller the ratio, the fewer Cu atoms exist around the base point Cu atom.

The research objects of the simulated tensile process were the models obtained after the aging of Fe-3.5Cu-0V, Fe-3.5Cu-0.1V and Fe-3.5Cu-0.2V alloys for 200 ns. When the energy minimization of the alloy model obtained by aging was completed, the model was relaxed for 200 ps at 300 K under NVT ensemble. Then a series of unidirectional strains was imposed on the relaxed model along the Y axis under the NVE ensemble until the model broke. The tensile strain rate was 1 × 10^9^ s^−1^. In the simulation, free boundary condition was adopted in the Y-axis direction, and periodic boundary condition was used in the X and Z-axis direction.

## 3. Results and Discussion

### 3.1. Calculation of the Binding Energy of Cu–Cu and Cu–V and the Migration Energy Barrier of Vacancies 

In order to investigate the impact of vacancies on the diffusion and precipitation of Cu atoms in BCC-Fe, the binding energy of Cu–Cu and Cu–V in different neighboring states was firstly calculated using Equation (1). The binding energy of Cu–Cu and Cu–V from the first neighbor (b1nn) to the fifth neighbor (b5nn) is shown in Table 1. The calculation results of binding energy in this paper are relatively consistent with the calculation results obtained by Zhang et al. [11], which proves the correctness of the simulation parameters selected in this paper. It can be seen from the table that in the b1nn and b2nn neighboring state, the binding energy of Cu–Cu and Cu–V is positive, indicating that Cu atoms and Cu atoms and vacancies are attracted to each other in this neighboring state. However, in the b1nn and b2nn neighboring state, the binding energy of Cu–Cu is less than that of Cu–V which means that in this neighboring state, the mutual attraction between Cu atoms and Cu atoms is smaller than that between Cu atoms and vacancies. Therefore, in BCC-Fe, Cu atoms tend to bind to vacancies rather than to Cu atoms themselves.

**Figure 3:**
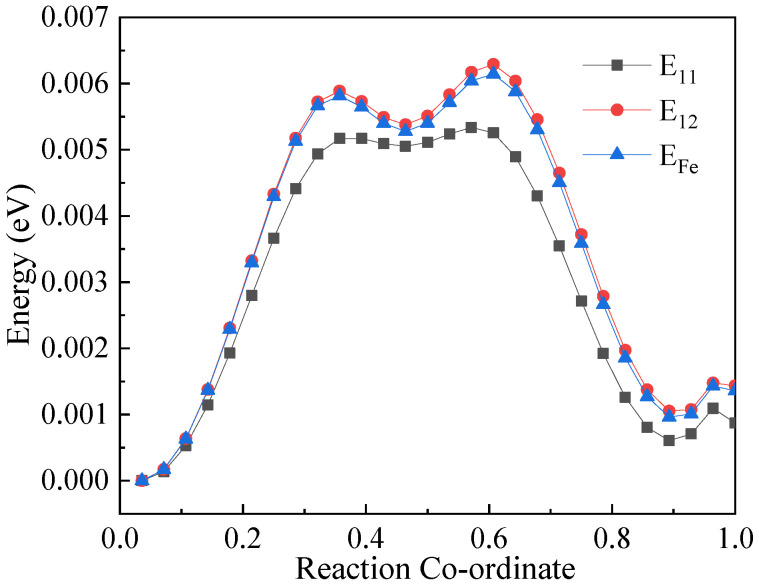
Migration energy barriers required for the vacancy to migrate along different paths. E_11_ represents the migration energy required for the exchange of positions between the Cu atoms and the vacancy; E_12_ represents the migration energy required for the vacancy to migrate from the first neighbor of the Cu atoms to the second neighbor of Cu atoms; E_Fe_ means the migration energy required for the position exchange between vacancy and Fe atom.

Subsequently, the NEB method was used to calculate the migration energy barrier of vacancies in BCC-Fe when migrating along different paths. The relative positions of the vacancies and Cu atoms is shown in Figure 2. E_11_ represents the migration energy required for the exchange of positions between the Cu atoms and the vacancy, and E_12_ represents the migration energy required for the vacancy to migrate from the first neighbor of the Cu atoms to the second neighbor of Cu atoms. E_Fe_ means the migration energy required for the position exchange between vacancy and Fe atom, and the migration energy barrier of the vacancy when migrating along different paths is shown in Figure 3. It can be seen from the figure that when the migration path of the vacancy is far away from the Cu atom, the energy required for migration is the largest. The migration energy required for the position exchange between Cu atoms and the vacancy is the smallest, which is less than that required for the position exchange between the vacancy and Fe atom. Combining the calculation results of binding energy, it can be seen that Cu atoms tend to bind to vacancies. When the vacancy is combined with Cu atoms, exchange of position occurs easily. Therefore, a preliminary conclusion was obtained that vacancies will promote the diffusion and precipitation of Cu atoms in BCC-Fe.

### 3.2. The Effect of the Concentration of Vacancies on the Diffusion and Precipitation of Cu Atoms in BCC-Fe

**Figure 4:**
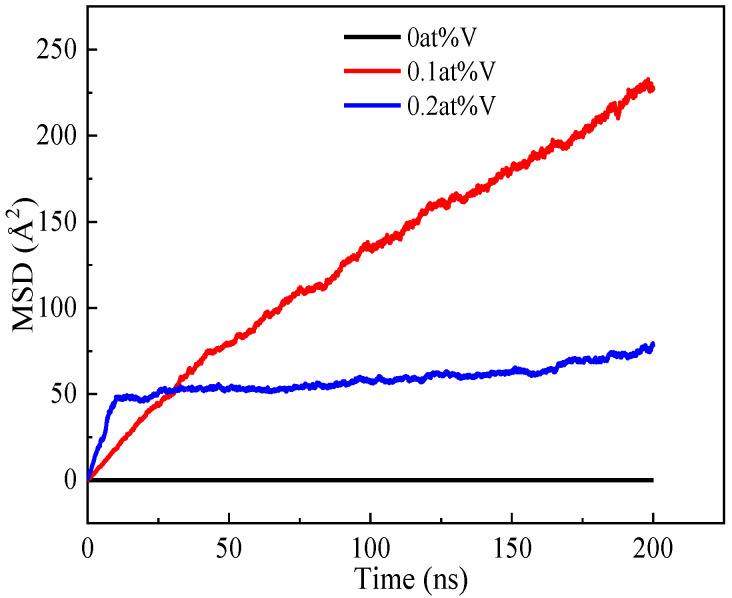
The relationship between MSD (mean-squared displacement) of Cu atoms and time in the Fe-Cu-0V, Fe-3.5Cu-0.1V and Fe-3.5Cu-0.2V alloys after aging at 1050 K for 200 ns.

In order to further verify that the presence of vacancies will improve the diffusion and precipitation ability of Cu atoms and reveal how the vacancy concentration affects the diffusion and precipitation of Cu atoms, the diffusion coefficients of Cu atoms in the Fe-3.5Cu-0V, Fe-3.5Cu-0.1V and Fe-3.5Cu-0.2V alloys during the aging process were calculated. Figure 4 shows the relationship between the MSD of Cu atoms and aging time in the Fe-3.5Cu-0V, Fe-3.5Cu-0.1V and Fe-3.5Cu-0.2V alloys when the alloys aged at 1050 K for 200 ns. As shown in the figure, when there are no vacancies in the Fe-3.5Cu alloy, the MSD of Cu atoms in the alloy is almost 0, indicating that there is almost no migration of Cu atoms in the alloy under this condition; when there is 0.1at%V in the alloy, the MSD of Cu atoms shows a good linear relationship with the aging time; when there exists 0.2at%V in the alloy, the relationship between the MSD of Cu atoms and the aging time can be divided into two stages: from 0–10 ns, the MSD of Cu atoms has a good linear relationship with the aging time, and at this stage, the slope of the relationship between the MSD of Cu atoms and the aging time is significantly greater than the slope of the relationship between the MSD of Cu atoms and the aging time in the alloy containing 0.1at%V. From 10-200 ns, the MSD of Cu atoms increases slightly with the extended aging time. According to Equation (4), the diffusion coefficient of Cu atoms in the Fe-3.5Cu alloy was calculated under different vacancy concentrations. When the alloy has no vacancies, 0.1 at% vacancies and 0.2 at% vacancies respectively, the diffusion coefficients of Cu atoms are, respectively, 2.37 × 10^−14^, 1.78 × 10^−8^ and 2.65 × 10^−9^ cm^2^·s^−1.^ The calculation results are relatively consistent with the calculation results obtained by Lv et al. [23] (when the Fe-1.0at%Cu alloy contains 0.1at% vacancies, the diffusion coefficient of Cu atom is 8.39 × 10^−8^ cm^2^·s^−1^). The calculation results of the diffusion coefficient of Cu atoms showed that when vacancies exist in the alloy, the diffusion coefficient of Cu atoms will increase significantly. This result further confirms that the presence of vacancies will improve the diffusion and precipitation ability of Cu atoms. However, in the alloy containing 0.2 at% vacancies, the diffusion coefficient of Cu atoms is one order of magnitude smaller than that of Cu atoms in the alloy containing 0.1 at% vacancies, suggesting that the diffusion and precipitation ability of Cu atoms does not always increase with the increasing vacancy concentration in the alloy.

**Figure 5:**
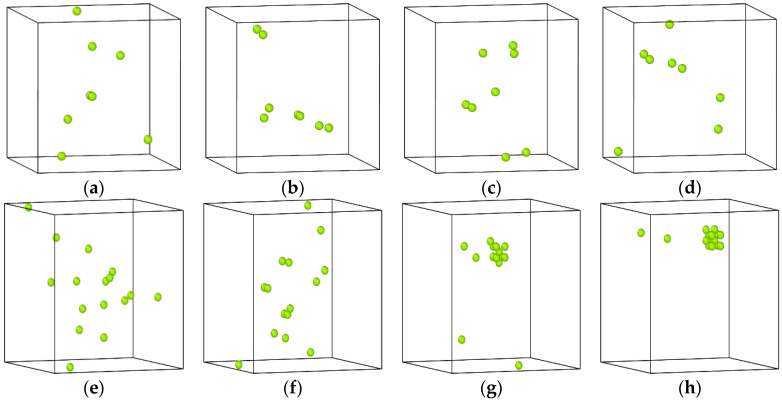
Distribution morphology of vacancies in the system when Fe-3.5Cu-0.1V and Fe-3.5Cu-0.2V alloys are aged for different times. For Fe-3.5Cu-0.1V alloy where (**a**–**d**) are 0 ns, 40 ns, 132 ns and 186 ns, respectively, and Fe-3.5Cu-0.2V alloy where (**e**–**h**) are 0 ns, 5 ns, 10 ns, and 132 ns, respectively.

In order to find the reason why the diffusion coefficient of Cu atoms in the alloy with 0.2 at% vacancies is smaller than that in the alloy containing 0.1 at% vacancies, the movement trajectories of vacancies inside the system were tracked and observed in the aging process of Fe-3.5Cu-0.1V and Fe-3.5Cu-0.2V alloys. When the Fe-3.5Cu-0.1V and Fe-3.5Cu-0.2V alloys were aged for different times, the vacancy distribution in the system is shown in Figure 5. It can be seen that when the alloy contains 0.1 at% vacancies, the vacancies appear in pairs in the system during the aging process, and the distribution of vacancies has changed, indicating that the vacancies frequently migrate during the aging process. In the alloy containing 0.2 at% vacancies, the vacancies also appear in pairs at the initial stage of aging, and their distribution has also changed. However, after 10 ns of aging, clusters occur in the pairs of vacancies in the original system. Marian et al. [24] also reported that clusters of vacancies will occur if the concentration of vacancies in the alloy exceeds a certain value. After the clusters of vacancies are generated, the number of freely migrating vacancies in the system decreases sharply, and is less than the number of freely migrating vacancies in the Fe-3.5Cu-0.1V alloy. From the calculation of the binding energy and migration energy in the previous section, we know that the diffusion and migration of Cu atoms are completed by exchanging positions with vacancies. Therefore, in the alloy containing 0.2 at% vacancies, the diffusion coefficient of Cu atoms is smaller than that in the alloy containing 0.1 at% vacancies.

### 3.3. The Impact of Vacancy Concentration on the Growth of Cu Precipitates

**Figure 6:**
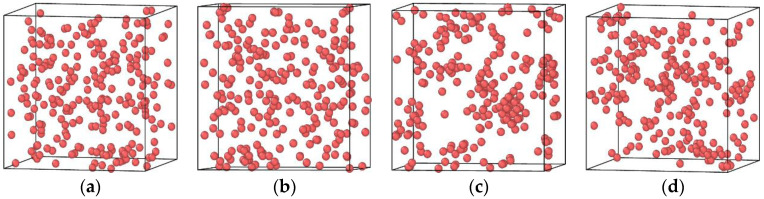
Cu atom distribution in the Fe-3.5Cu alloy before aging and after aging at 1050 K for 200 ns when the vacancy content is different (**a**) before aging (**b**) without vacancies (**c**) with 0.1 at% vacancies (**d**) with 0.2 at% vacancies.

Figure 6 shows the distribution of Cu atoms in the Fe-3.5Cu alloy before and after aging when the vacancy content varies. It can be seen from the figure that before the alloy is aged and after the alloy without vacancies is aged for 200 ns, the Cu atoms in the Fe-3.5Cu alloy are all dispersed, with no clusters generated. By observing the distribution of Cu atoms in the aged alloy with 0.1 at% vacancies and 0.2 at% vacancies, it can be seen that no matter whether the vacancy content is 0.1 at% or 0.2 at%, obvious clusters of Cu atoms occur in the aged system, which further confirms that the existence of vacancies will promote the diffusion and precipitation of Cu atoms in Fe–Cu alloys. Figure 6c,d also show that in the alloy containing 0.1 at% vacancies, the size of Cu clusters produced after aging is significantly larger than that in the aged alloy containing 0.2 at% vacancies; when the alloy contains 0.2 at% vacancies, the number of Cu clusters produced after aging is more than that in the aged alloy with 0.1 at% vacancies. In the following part, the reasons for this phenomenon are explained through the observation and analysis of Figure 4 and Figure 5.

It can be seen from Figure 5 that at the initial stage of aging of the alloy containing 0.2 at% vacancies, the vacancies can move freely, and there are no stable clusters of vacancies. As shown in Figure 4, when the aging time is less than 10 ns, the slope of the relationship between the MSD of Cu atoms in the alloy with 0.2 at% vacancies and aging time is significantly greater than that of the relationship between the MSD of Cu atoms in the alloy containing 0.1 at% vacancies and aging time. This is the reason why at the early stage of aging, the diffusion ability of Cu atoms in the alloy with 0.2 at% vacancies is greater than that in the alloy with 0.1 at% vacancies. Therefore, at the initial stage of aging, the rate of Cu precipitation in the alloy containing 0.2 at% vacancies is greater than that in the alloy containing 0.1 at% vacancies. However, with the extended aging time, clusters of vacancies occur in the alloy containing 0.2 at% vacancies, and the diffusion ability of Cu atoms in the alloy is significantly reduced. Therefore, with the extension of aging time, the growth rate of Cu precipitates in the alloy containing 0.2 at% vacancies is lower than that in the alloy containing 0.1 at% vacancies. This can explain why after 200 ns of aging, although the size of the Cu precipitates in the alloy with 0.2 at% vacancies is smaller than that in the alloy with 0.1 at% vacancies, the number of Cu precipitates in the alloy with 0.2 at% vacancies is more than that in the alloy with 0.1 at% vacancies.

**Figure 7:**
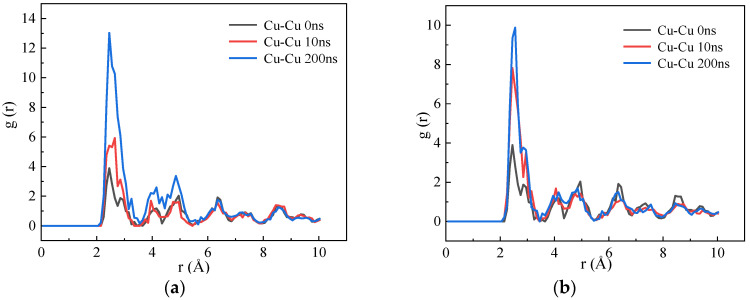
The radial distribution function of Fe-3.5Cu alloy with 0.1 at% and 0.2 at% vacancies at different aging time (**a**) Fe-3.5Cu-0.1V alloy (**b**) Fe-3.5 Cu-0.2V alloy.

In order to further illustrate the impact of vacancy concentration on the growth of Cu precipitates in Fe–Cu alloy, the radial distribution function was used to analyze the growth of Cu precipitates during the aging process. The Cu–Cu radial distribution function of the Fe-3.5Cu alloy with different vacancy concentrations after being aged for different time is shown in Figure 7. It can be seen from the figure that for the alloy containing 0.1 at% vacancies, the peak value of the first peak of the radial distribution function after 10 ns of aging increases by 2 compared to that before aging. For the alloy system containing 0.2 at% vacancies, the peak value of the first peak of the radial distribution function after 10 ns of aging is increased by 4.2 compared with that before aging. The figure also reveals that for the alloy system containing 0.1 at% vacancies, the peak value of the first peak of the radial distribution function after 200 ns of aging is increased by 7.5 compared with that when the alloy is aged for 10 ns. Regarding the alloy containing 0.2 at% vacancies, the peak value of the first peak of the radial distribution function after 200 ns of aging is increased by 2.2 compared with that when the alloy is aged for 10 ns. It can be seen that in the early stage of aging, the rate of Cu precipitation in the alloy containing 0.2 at% vacancies is faster than that in the alloy containing 0.1 at% vacancies. However, with the extension of the aging time, the growth rate of the Cu precipitates in the alloy containing 0.2 at% vacancies is slower than that in the alloy containing 0.1 at% vacancies. Moreover, it can be seen from the figure that in the alloy system containing 0.1 at% vacancies, the values of the second and third peaks of the radial distribution function after 200 ns of aging show a significant increase compared to those when the alloy is aged for 10 ns. However, in the alloy system containing 0.2 at% vacancies, the values of the second and third peaks of the radial distribution function after 200 ns of aging have no significant increase compared to those when the aging time is 10 ns. This phenomenon further confirms that when the aging time is longer than 10 ns, the growth rate of Cu precipitates in the alloy with 0.1 at% vacancies is much faster than that in the alloy with 0.2 at% vacancies.

### 3.4. The Influence of Cu Precipitates on the Tensile Properties of Fe–Cu Alloy

**Figure 8:**
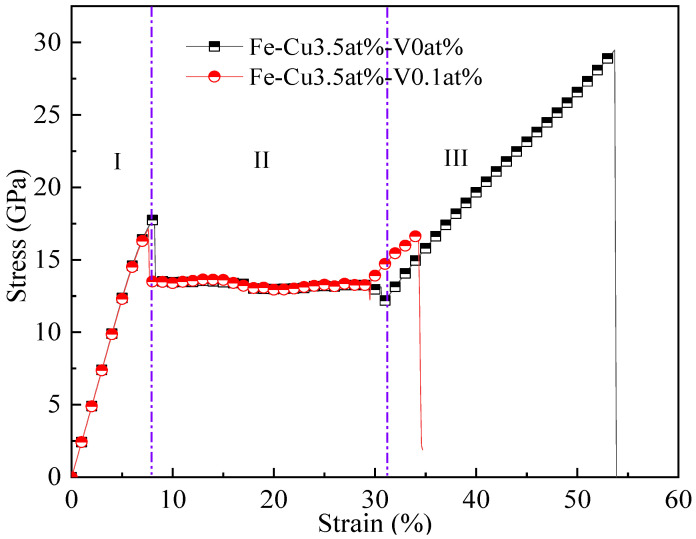
Stress–strain diagram of the Fe-3.5at%Cu-0at%V and Fe-3.5at%Cu-0.1at%V alloys after aging at 1050 K for 200 ns. I is the elastic deformation stage; II is the plastic deformation stage; III is the strain hardening stage.

Figure 8 shows the stress–strain curves of Fe-3.5Cu-0V and Fe-3.5Cu-0.1V alloys after aging at 1050 K for 200 ns. First, the stress–strain curve of the aged Fe-3.5Cu-0V alloy was analyzed. It can be seen from the figure that after the aging of the Fe-3.5Cu-0V alloy, the stress–strain curve obtained by stretching can be divided into three parts: The first part is where the strain is 0–8.2%. At this stage, the stress increases with the increase in strain, and there is basically a linear relationship between strain and stress, which is an elastic deformation stage; the second is where the strain is 8.2–31.5%. At this stage, the stress does not increase with the increase in strain, and the stress value fluctuates around 13 GPa. This is the plastic deformation stage; the third part is where the strain is 31.5–53.7%. At this stage, the crystal recovers its ability to resist deformation, and the stress begins to increase as the strain rises. This is the strain hardening stage. It can be seen from the figure that the stress–strain curve obtained by stretching the aged Fe-3.5Cu-0.1V alloy can also be divided into three parts: elastic deformation, plastic deformation and strain hardening. However, by comparing the stress–strain curves obtained by stretching the aged Fe-3.5Cu-0V and Fe-3.5Cu-0.1V alloys, it was found that the yield strength of the aged Fe-3.5at%Cu-0.1at%V alloy is slightly lower than that of the Fe-3.5Cu-0V alloy after aging, and the strain hardening strength is significantly reduced. The analysis in the previous section revealed that after the aging of Fe-3.5Cu-0V, the Cu atoms in the system are dispersed, with no Cu precipitates produced. However, after the aging of the Fe-3.5Cu-0.1V alloy, obvious clusters of Cu atoms appear in the system. Therefore, it can be concluded that the decreased yield strength and weakened strain hardening strength of the aged Fe-3.5Cu-0.1V alloy are caused by the Cu precipitates.

**Figure 9:**
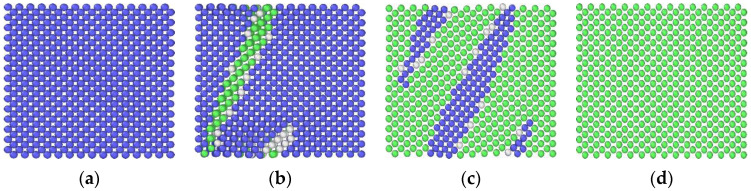
The evolution of the internal crystal structure of the aged Fe-3.5Cu-0V alloy (**a**) 0%-8.2% strain (**b**) 8.3% strain (**c**) 27% strain (**d**) 31.5–53% strain, blue part represents BCC structure; green part denotes FCC structure; white part represents other structures.

In order to explore the mechanism of the changes in the tensile properties of the Fe-3.5Cu-0.1V alloy caused by the Cu precipitates, the internal crystal structure and defect evolution in the system during the tensile process of the aged Fe-3.5Cu-0V and Fe-3.5Cu-0.1V alloys were observed and analyzed. Figure 9 shows the internal crystal structure evolution during the tensile process of aged Fe-3.5Cu-0V alloy. It can be seen from Figure 9a that when the strain is 0–8.2%, the internal crystal structure of the system is BCC; when the strain exceeds 8.2%, as shown in Figure 9b, structural phase change occurs, with the internal crystal structure in some areas changing from BCC to FCC (Face-Centered Cubic). Then, as the strain continues to increase, the area where the structural phase change occurs in the system is expanded; when the strain reaches 31.5%, as shown in Figure 9d, the crystal structure of all areas in the system has changed from BCC to FCC. After that, the strain continues to increase until the crystal breaks, and the crystal structure no longer changes. Li et al. [25] also found a similar structural phase change phenomenon when studying the tensile properties of tungsten. It can be seen that when the strain exceeds 8.2%, structural phase change will appear in the crystal to resist the action of the external load, resulting in the phenomenon that the strain increases while the stress value fluctuates around 13 GPa. Therefore, the phase change of the crystal structure during the tensile process is the reason for the plastic deformation of the Fe-3.5Cu-0V alloy during the stretching process.

**Figure 10:**
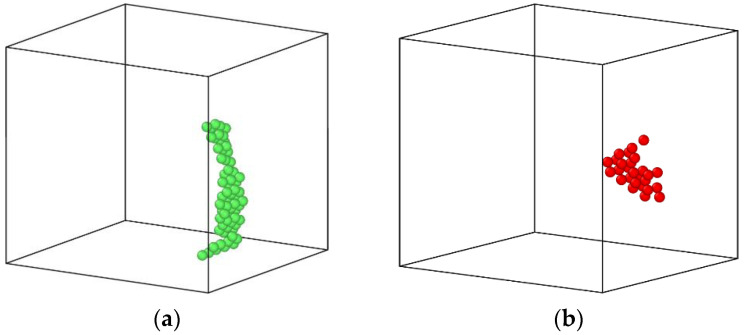
The initial area of structural phase change and the Cu precipitate with the maximum size in the Fe-3.5Cu-0.1V alloy during the tensile process (**a**) The area of structural phase change at a strain of 7.6% (**b**) Cu precipitate.

It can be seen from the stress–strain curve that the plastic deformation of the aged Fe-3.5Cu-0.1V alloy occurs earlier than that of the aged Fe-3.5Cu-0V alloy, and the yield strength is reduced. It can also be seen from the analysis in the previous section that the plastic deformation in the stretching process is caused by the phase change of the crystal structure. Therefore, it can be concluded that the aged Fe-3.5Cu-0.1V alloy has a structural phase change earlier than the aged Fe-3.5Cu-0V alloy during the tensile process. In order to reveal the reason why the aged Fe-3.5Cu-0.1V alloy has an early structural phase change during stretching compared to the aged Fe-3.5Cu-0V alloy, the initial area of structural phase change and the Cu precipitate with the maximum size in the tensile process of the aged Fe-3.5Cu-0.1V alloy were separately displayed, as shown in Figure 10. It can be seen from the figure that when the strain reaches 7.6%, the structural phase transition appears in the Fe-3.5Cu-0.1V alloy system. It can also be observed from the figure that the structural phase change occurs first in the area where the Cu precipitate is. Therefore, it can be concluded that the aged Fe-3.5Cu-0.1V alloy has an earlier plastic deformation than the aged Fe-3.5Cu-0V alloy, and the yield strength is decreased, which are caused by the early phase transition of the crystal structure triggered by the Cu precipitate.

**Figure 11:**
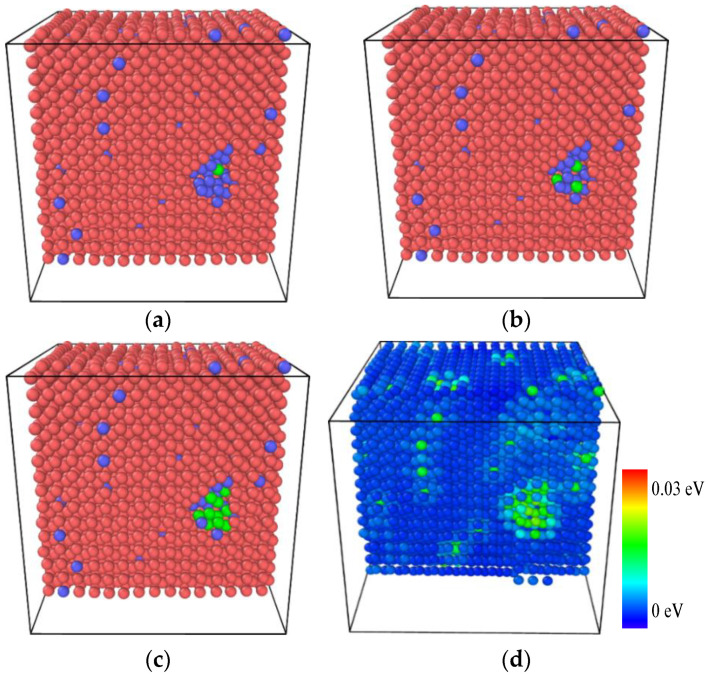
The internal defect evolution and atomic energy distribution in the aged Fe-3.5Cu-0.1V alloy during the tensile process, (**a**–**c**) respectively show the vacancy distribution at 31% 5 strain, 32.5% strain and 34% strain. (**d**) When the strain is 34%, the atoms are colored according to the atomic energy, Red for Fe atoms; blue for Cu atoms; green for vacancies.

Figure 11 exhibits the internal defect evolution and atomic energy distribution in the aged Fe-3.5Cu-0.1V alloy during the stretching process, and the alloy model is displayed in cross-section to facilitate observation and analysis. It can be seen from Figure 11a–c that when the strain is 30%, that is, after the plastic deformation stage caused by the structural phase change, if the strain is continuously increased, the vacancy defects will be generated in the Cu precipitate first, and then the number of vacancy defects will increase with the increase in strain until the crystal breaks. In Figure 11d, the atoms are colored according to the energy of the atoms in the crystal. It can be observed that the energy of atoms around the vacancy defect is significantly higher than the energy of atoms at other positions in the crystal, suggesting that the appearance of vacancies results in serious distortion of the surrounding lattice, and leads to increased energy of atoms around the vacancies, thus weakening the structural stability of this area. Therefore, compared with the aged Fe-3.5Cu-0V alloy, the aged Fe-3.5Cu-0.1V alloy will fracture earlier during the stretching process, thus reducing the strain hardening strength.

## 4. Conclusions

1. The presence of vacancies in the Fe-3.5Cu alloy will improve the diffusion and precipitation ability of Cu atoms, and the diffusion and precipitation ability of Cu atoms will first increase and then reduce with the increase in vacancies in the alloy. When the aging temperature is 1050 K, and the alloy contains no vacancies, 0.1 at% and 0.2 at% vacancies respectively, the diffusion coefficient of Cu atoms is 2.37 × 10^−14^, 1.78 × 10^−8^ and 2.65 × 10^−9^ cm^2^·s^−1^ respectively.
2. During the aging process, the existence of vacancies in the Fe-3.5Cu alloy will stimulate the generation of Cu precipitates, and the concentration of vacancies has an important impact on the growth of Cu precipitates. When there are no vacancies in the alloy, aging will not produce Cu precipitates. In the alloy containing 0.1at% vacancies, the size of the Cu precipitates produced by aging will be larger than that of Cu precipitates generated in alloy with 0.2at% vacancies, but the amount of Cu precipitates produced by aging is less than that in the alloy with 0.2at% vacancies.
3. Compared to the aged Fe-3.5Cu-0V alloy, during the tensile process, the aged Fe-3.5Cu-0.1V alloy will have structural phase transition earlier and vacancy defects appear in the system, so that the yield strength of the aged Fe-3.5Cu-0.1V alloy is slightly lower and the strain hardening strength is significantly reduced compared with that of the aged Fe-3.5Cu-0V alloy.

## Figures and Tables

**Table 1:** Binding energy (eV) of Cu–Cu and Cu–V in different neighboring methods in BCC-Fe.

Cu–Cu Neighboring Method	Present	Zhang [11]	Cu–V Neighboring Method	Present	Zhang [11]
$E_{b1nn}^{\mathrm{Cu}-\mathrm{Cu}}$	0.079	0.08	$E_{b1nn}^{\mathrm{Cu}-V}$	0.093	0.10
$E_{b2nn}^{\mathrm{Cu}-\mathrm{Cu}}$	0.078	0.08	$E_{b2nn}^{\mathrm{Cu}-V}$	0.085	0.09
$E_{b3nn}^{\mathrm{Cu}-\mathrm{Cu}}$	−0.001	-	$E_{b3nn}^{\mathrm{Cu}-V}$	−0.204	-
$E_{b4nn}^{\mathrm{Cu}-\mathrm{Cu}}$	−0.003	-	$E_{b4nn}^{\mathrm{Cu}-V}$	−0.252	-
$E_{b5nn}^{\mathrm{Cu}-\mathrm{Cu}}$	−0.019	-	$E_{b5nn}^{\mathrm{Cu}-V}$	−0.403	-

## Data Availability

All the data is available within the manuscript.
